# Electro-Spun P(VDF-HFP)/Silica Composite Gel Electrolytes for High-Performance Lithium-Ion Batteries

**DOI:** 10.3390/ma17205083

**Published:** 2024-10-18

**Authors:** Wen Huang, Caiyuan Liu, Xin Fang, Hui Peng, Yonggang Yang, Yi Li

**Affiliations:** Jiangsu Key Laboratory of Advanced Functional Polymer Materials, Department of Polymer Science and Engineering, College of Chemistry, Chemical Engineering and Materials Science, Soochow University, Suzhou 215123, China

**Keywords:** lithium-ion battery, composite material, gel polymer electrolyte, electrospinning, sol-gel preparation

## Abstract

This work presents a facile way to fabricate a polymer/ceramics composite gel electrolyte to improve the overall properties of lithium-ion batteries. Lithium salt-grafted silica was synthesized and mixed with P(VDF-HFP) to produce a nanofiber film by the electrostatic spinning method. After coating a layer of SiO_2_ onto the surface of nanofibers through a sol-gel method, a composite nanofiber film was obtained. It was then immersed in plasticizer until saturation to make a composite gel electrolyte film. Electrochemical test results showed that the obtained gel electrolyte film shows high thermal stability (~450 °C), high ionic conductivity of 1.3 × 10^−3^ S cm^−1^ at 25 °C and a lithium-ion transference number of 0.58, and superior cycling stability, providing a new direction for manufacturing secondary batteries with higher safety and performance.

## 1. Introduction

With increasing global environmental degradation and dwindling energy reserves, modern society urgently requires energy storage devices that are both valid and clean. Lithium-ion batteries (LIBs) are not only lightweight, but also have long cycle life, high energy density, and are environmentally friendly [1]; therefore, they are applied extensively in the realm of portable electronics, electric cars, and various devices [2,3,4,5].

Most commercial LIBs utilize liquid electrolytes as the medium for ion transport, primarily because of their excellent ion conduction and ability to maintain stable contact with various electrodes. Liquid electrolytes usually consist of solutions of lithium salt dissolved in organic solvents like ethylene carbonate (EC), dimethyl carbonate (DMC), and diethyl carbonate. They are volatile and flammable, and are prone to cause safety problems such as leakage and fire, which are usually aroused by lithium dendrite growth [6]. Lithium dendrite is formed by uneven lithium nucleation on anode surfaces or unstable lithium-ion ionic currents. Such uneven lithium nucleation and growth will further bring about anode chalking, continuous electrolyte depletion, and even puncture of the separator [7,8,9]. Switching from liquid electrolytes to solid electrolytes represents a significant approach to addressing the aforementioned challenges [10]. In the case of solid inorganic electrolytes, although they have high ionic conductivity, their practical application is limited by their brittleness and bad charge transfer due to poor electrode/electrolyte contact [11]. Polymer electrolytes can be categorized into two groups in light of their composition: solid polymer electrolytes and gel polymer electrolytes (GPEs, quasi-solid electrolytes). In contrast to solid inorganic electrolytes, solid polymer electrolytes offer numerous benefits such as straightforward synthesis, a low mass per volume, and affordability. Additionally, their favorable flexibility aids in accommodating the volume change of the electrode when operating, which leads to mitigating the interfacial resistance and enhancing the interfacial stability between electrode and electrolyte. Nonetheless, due to the lithium-ion transport in polymer electrolytes occurring through the movement of chain segments, the ionic conductivity at ambient temperature for solid polymer electrolytes is significantly lower than what is necessary for practical use [12]. GPEs, composed of a polymer framework, organic solvent, and electrolyte salt, combine the benefits of liquid and solid electrolytes, and meanwhile serve the dual function of electrolyte and separator [13,14]. The polymer network prevents electrolyte leakage and provides high mechanical strength, and the solvent liquid not only encapsulates the polymer chain segments and improves ion conduction, but also improves the interfacial compatibility between the GPEs and the electrode. Therefore, GPEs are considered to be the most promising electrolytes for LIBs.

Commonly used polymers as the main body of GPEs are polyvinylidene fluoride, poly(vinylidene fluoride-co-hexafluoropropylene) (P(VDF-HFP)), polyethylene oxide, polyacrylonitrile, and poly(propylene carbonate) [15,16,17,18]. Compared with commercial polyolefin separators, P(VDF-HFP) not only has good chemical stability, but also has higher liquid absorption and better mechanical properties, endowing batteries with increased energy densities, accelerated reaction rates, and enhanced safety [19,20,21]. For example, Jie et al. used N-methyl-2-pyrrolidone (NMP) to fabricate flexible P(VDF-HFP)-based GPEs, and they had a superior ionic conductivity of 7.2 × 10^−4^ S cm^−1^ at 70 °C, which demonstrated favorable interfacial compatibility with the lithium anode and successfully inhibited the development of lithium dendrite [20]. Moreover, electro-spun P(VDF-HFP)-based GPEs have been reported as benign separators in LIBs because the electrostatic spinning technique can produce porous films with fibrous structures characterized by tunable composition, high porosity, and chemical stability [22,23].

However, GPEs are largely limited by insufficient mechanical strength and instability under high temperature. To address these challenges, a valid approach involves incorporating inorganic nanoparticles into the polymer matrices. These additives have the ability to suppress crystallization and improve the mechanical properties of GPEs [24,25]. Utilizing nanoparticles like silica, titanium dioxide, and zirconium dioxide for enhancement can notably increase the mechanical property, heat stability, and ionic conductivity of GPEs [26,27,28,29,30,31]. To illustrate, Na et al. chemically grafted silica nanoparticles onto a porous polyethylene separator, providing superior ionic conductivity of 8.4 × 10^−4^ S cm^−1^ to conventional physical coatings of polymer composites based on ceramic particles [31]. However, this method also posed a problem in that the nanoparticles added to the surface of the separator may be dislodged during subsequent processing, leading to uneven current densities on the electrodes during cycling and accelerated battery degradation.

Moreover, the majority of LIB systems typically function as dual-ionic conductors, allowing for the mobility of both cations and anions. This conductivity arises from the dissolution of electrolyte salts within the polymer matrix. Because of concentration polarization, the performance of a dual-ionic conductor battery is degraded, including an increase in internal impedance, voltage loss, and so on [32]. Single-ion conducting polymer electrolytes significantly alleviate lithium-ion concentration polarization by having anions attached to the polymer backbone, enabling only lithium ions to move. Lithium ions are the only charge conduction carrier, so the lithium-ion transference number can approach 1.

Considering the above-mentioned inherent shortcomings of GPEs, the single modification way seems inadequate to improve the comprehensive properties of GPEs. Thus, in this work, we present a facile fabrication way comprising an electro-spun polymer nanofiber matrix, a doped single-ion conductor, and composite ceramics. Firstly, a lithium salt-grafted silica was synthesized, then it was mixed with P(VDF-HFP) to make nanofiber film (named EPS) via electrostatic spinning. Secondly, a layer of SiO_2_ was coated onto the surface of nanofibers by a sol-gel method to improve their mechanical properties, and the obtained composite film was named EPSS. Finally, EPS and EPSS served dual roles both as separator and as scaffold to make gel electrolyte films. Their electrochemical characteristics were evaluated, and excellent thermal stability, a high electrochemical stability window, and superior ionic conductive behavior were achieved, indicating potential application for secondary batteries.

## 2. Materials and Methods

### 2.1. Materials

Anhydrous ethanol (>99.7%), dichloromethane (>99.5%), and ammonia (NH_3_·H_2_O, 25 wt%) were provided by Sinopharm Chemical Reagent Co., Ltd. (Shanghai, China). Poly(vinylidene fluoride-co-hexafluoropropylene) (*M*_w_ = 455,000), lithium hydroxide, cetyltrimethylammonium bromide, and N,N-dimethy-l-formamide (DMF, >99.9%) were supplied by Aladdin. (Shanghai, China). Tetraethyl orthosilicate (TEOS, >99.5%) was provided by Jiangsu Qiangsheng Functional Chemical Co., Ltd. (Suzhou, China). N-hexanol and hydrochloric acid (HCl) were bought from Shanghai Lingfeng Chemical Reagent Co., Ltd. (Shanghai, China). Acetonitrile, 3-chloropropanesulfonyl chloride and Fmoc-L-Ala were purchased from Shanghai Meryer Biochemical Technology Co., Ltd. (Shanghai, China). Ethylene carbonate and dimethyl carbonate were produced by Guotai Huarong Chemical New Materials Co., Ltd. (Suzhou, China). Trifluoromethanesulfonamide, lithium bis(trifluoromethane sulfonyl) imide (LITFSI) and LiFePO_4_ were supplied by Shanghai Macklin Biochemical Technology Co., Ltd. (Shanghai, China). Cetylpyridinium chloride (CPC) and tris(hydroxymethyl)aminomethane were produced by Tixiai (Shanghai, China) Chemical Industry Development Co., Ltd. (Shanghai, China). Lithium hydride was bought from Shanghai Jingpure Biochemical Technology Co., Ltd. (Shanghai, China). All materials were used as received without further purification.

### 2.2. Preparation of Composite Gel Electrolyte Films

#### 2.2.1. Synthesis of Lithium Salt-Grafted Silica

Lithium 3-chloroproanesulfonyl(trifluoromethanesulfonyl)imide (LiCPSI) was synthesized according to the literature [33].

Mesoporous SiO_2_ was prepared as follows: 200 mg of CPC and 18.4 mg of Fmoc-L-Ala were dissolved in 500 mL deionized water. Afterwards, 3 mL of concentrated NH_3_·H_2_O was introduced into the mixture, which was then stirred at 40 °C for 30 min. Thereafter, 148 μL of n-hexanol was added to the mixture followed by the addition of 6 mL TEOS. Then, 10 s later, the stirring was halted, and the solution gradually turned turbid and white. After letting the solution stand for 36 h, a suspension containing white flocculent clusters was obtained through supramolecular co-assembly of CPC and TEOS. The suspension was subjected to filtration, cleaned using ethanol, and dried at 70 °C for 6 h, yielding a white cotton-like powder. Finally, the powder was loaded into a tube furnace under nitrogen atmosphere and subjected to calcination at 700 °C for 6 h to eliminate the remaining template agent CPC, yielding the white mesoporous SiO_2_ powder.

The grafting of lithium salts was performed using the Williamson reaction according to the reference [34]. A total of 500 mg of SiO_2_ powder was dispersed in 100 mL of DMF within a dry three-necked flask. Under nitrogen protection, 30 mg of lithium hydride was introduced and the mixture was stirred at 60 °C for 3 h. Upon the addition of 100 mg of LiCPSI, the reaction temperature was increased to 90 °C. The mixture was stirred for 48 h. Thereafter, 50 mL of deionized water was introduced into the flask, and the solution was stirred at ambient temperature for 2 h. The mixture was filtered and washed with deionized water and ethanol, respectively, then dried at 80 °C for 24 h to acquire black lithium salt-grafted silica solid powder, which is denoted as SL.

#### 2.2.2. Preparation of Nanofiber Films and Composite Gel Electrolytes

SL (5, 10 or 15 wt%) and P(VDF-HFP) were added to DMF to make a spinning solution, in which the solid content was 0.22 g mL^−1^. After stirring at 55 °C for 4 h, a homogeneous spinning solution was formed. SL-doped P(VDF-HFP) film was prepared by the electrostatic spinning technique. The specific parameters were as follows: positive voltage was 9 kV, negative voltage was −5 kV, receiving distance was 15 cm, and pushing rate was 0.1 mm s^−1^. The collected nanofiber film (denoted as EPS) was dried at 70 °C overnight.

**Figure 1:**
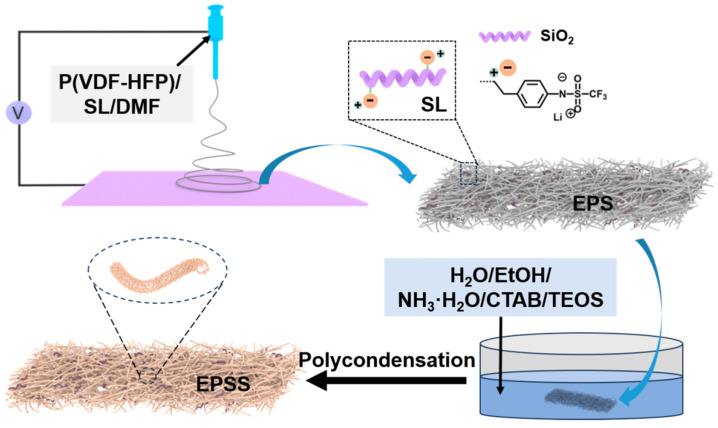
Illustration of the fabrication process of EPS and EPSS composite nanofiber films.

At 40 °C, 400 mg of cetyltrimethylammonium was added to 400 mL of ethanol/deionized water (1:100 *v*/***v***) mixture. A total of 50 mg of EPS film was immersed in this mixture for 2 min. Following this, 1 mL of TEOS and then 1 mL of concentrated NH_3_·H_2_O were introduced to the mixture dropwise, then the mixture was left to sit at 40 °C for 8 h. After washing several times using deionized water, the film was then refluxed in ethanol for 12 h to eliminate the surfactant, and dried at 70 °C for 24 h. The ultimately obtained film was denoted as EPSS. The fabrication process of EPS and EPSS films are illustrated in Figure 1.

EPS and EPSS films were cut into discs of 16 mm diameter and soaked in liquid plasticizer (EC/DMC = 1/1, *v*/***v***) until absorption saturation to make gel electrolyte films. When EPSS was immersed in electrolyte (0.2 M LiTFSI in EC/DMC = 1/1, *v*/***v***), EPSS-0.2 gel electrolyte film was obtained.

### 2.3. Methods and Characterizations

X-ray photoelectron spectroscopy (XPS) analyses were taken with EXCALAB 250 XI equipment from Thermo Fisher Scientific (Waltham, MA, USA). Transmission electron microscopy (TEM) images were collected with HT7700 equipment from HITACHI (Tokyo, Japan) at 200 kV. Field emission scanning electron microscopy (FE-SEM) was carried out with Regulus 8230 equipment from HITACHI (Tokyo, Japan) operating at 15 kV. Thermal gravity analysis (TGA) measurements were carried out with Thermal Analysis TG/DTA 6300 equipment from NSK LTD. (Tokyo, Japan) under a nitrogen atmosphere, with a heating rate of 10 °C min^−1^.

### 2.4. Battery Assembly and Electrochemical Tests

The electrochemical workstation CHI 660 E from Shanghai Chenhua Instrument Co., Ltd. (Shanghai, China) was utilized for electrochemical testing. For constant current charging and discharging tests, the LAND system CT 3002 A from Wuhan Bluetooth Electronics Co., Ltd. (Wuhan, China) was employed. Electrochemical impedance spectroscopy (EIS) measurements were performed across varying temperatures, employing an AC amplitude of 5 mV and a frequency between 100 kHz and 10 MHz. The potential of linear sweep voltammetry (LSV) was scanned from the open circuit voltage to 7.0 V (V vs. Li/Li^+^), and the speed was 5.0 mV per second. Symmetric charge/discharge cycles of the Li/Li battery were collected at ambient temperature using the Land CT 2001 A battery testing system, in order to assess the compatibility of the interface between the lithium metal electrode and the gel electrolyte film.

The assembly of the CR2016 button batteries took place within an argon-encased glove box. To construct a LiFePO_4_/Li battery, a slurry was prepared by mixing LiFePO_4_, acetylene black, and PVDF binder in a mass ratio of 8:1:1 in NMP. Afterwards, this slurry was coated onto an aluminum case, dried overnight, and cut into 14 mm diameter circles to form the cathode. The active material of the cathode had a mass of 1.8 mg. A lithium metal foil served as the counter electrode, and an electrolyte film cut into 16 mm diameter discs served as the separator. The liquid electrolyte comprised 0.2 M LiTFSI dissolved in a solution of DMC and EC in a 1:1 volume ratio. The cycling performance of the LiFePO_4_/Li battery at room temperature was evaluated at various current densities with 1C = 170 mAh g^−1^. Cyclic voltammetry tests and charge/discharge cycle tests were operated within the potential range of 2.5 to 4.2 V.

### 2.5. Calculations

The ionic conductivity (*σ*) was investigated by AC impedance analysis of a stainless steel (SS) symmetrical cell SS/SS:

(1) $$\sigma =\frac{l}{R_{b}S}$$

where *l* is the electrolyte film thickness, *R*_b_ is the electrolyte bulk resistance measured by EIS, and *S* is the cross-sectional area.

The activation energy (*E*_a_) for lithium-ion conduction can be calculated by the Arrhenius equation:

(2) $$\sigma =A\;exp\;(-\frac{E_{a}}{RT})$$

where *A* is the pre-exponential factor, *T* is the absolute temperature, and *R* is the Boltzmann constant.

The lithium-ion transference number (*t*_Li+_) at room temperature was determined using chronoamperometry and impedance spectroscopy measurements on a Li/Li symmetrical cell.

(3) $$t_{Li+}=\frac{I_{s}(\Delta V-I_{0}R_{0})}{I_{0}(\Delta V-I_{s}R_{s})}$$

where *I*_0_ and *I*_s_ are the initial and steady-state currents, *ΔV* is the polarization voltage (*ΔV* = 10 mV), and *R*_0_ and *R*_S_ are the interfacial resistances (Ω) before and after polarization, respectively.

## 3. Results and Discussion

**Figure 2:**
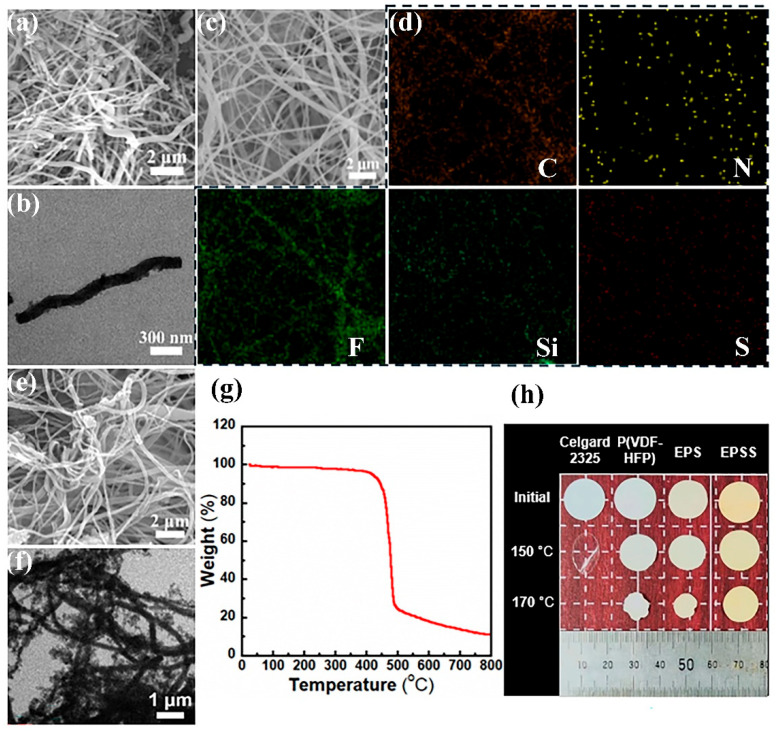
(**a**) FE-SEM and (**b**) TEM images of SL; (**c**) FE-SEM and (**d**) corresponding EDS mapping images of nanofibers in EPS; (**e**) FE-SEM and (**f**) TEM images of nanofibers in EPSS; (**g**) TGA curves of EPSS; (**h**) dimensional changes of Celgard 2325, P(VDF-HFP), EPS, and EPSS films after heat treatment at various temperatures. The SL content in EPS and EPSS is 10 wt%.

The obtained SL is shown in Figure 2a,b. They are helical nanofibers with lengths of tens of microns. By doping SL (5–15 wt%) into a P(VDF-HFP) spinning solution, an electro-spun P(VDF-HFP)/SL composite nanofiber film EPS was gained. The diameters of the fibers are about 300 nm, and the SL and matrix polymer fibers are entangled with each other and difficult to discern, as is illustrated by the FE-SEM and energy dispersive spectroscopy (EDS) mapping images in Figure 2c,d. The image of elements Si, N, and S indicated that SL is evenly distributed in the EPS film. After covering a layer of silica onto the P(VDF-HFP)/SL nanofibers by a sol-gel method, an EPSS nanofiber film was obtained, as is shown in Figure 2e,f. The TGA curve of EPSS under nitrogen atmosphere is shown in Figure 2g, indicating that it is thermally stable until 450 °C. The small mass loss between 20 °C and 450 °C originates from the decomposition of the organic moiety in the SL filler. In order to guarantee the safety of LIBs, the thermal dimensional stability of different separators was evaluated, as is depicted in Figure 2h. The commercial Celgard 2325 separator, P(VDF-HFP), EPS, and EPSS films were placed at 150 °C and 170 °C for 1 h. The picture indicates the EPSS film has the lowest dimensional change rate significantly, indicating the enhancement of the SiO_2_ layer for the safety of the separator.

**Figure 3:**
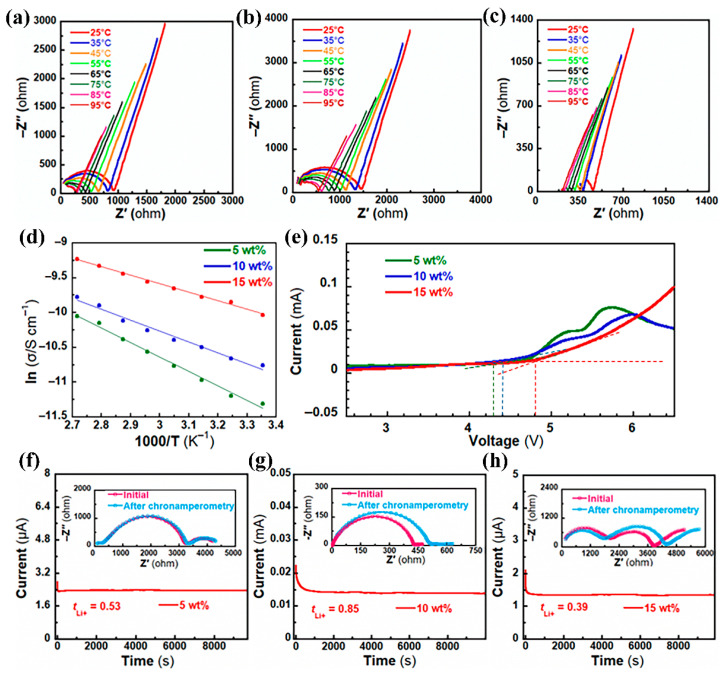
Electrochemical performance of EPS films with different SL doping amounts: (**a**–**c**) Nyquist plots in the frequency range of 10 Hz–100 KHz at different temperatures; (**d**) Arrhenius plots at different temperatures; (**e**) LSV curves; (**f**–**h**) chronoamperometry curves with a step voltage of 10 mV at room temperature (the insets are corresponding EISs before and after polarization).

When immersing EPS films in the organic solvent (plasticizer, EC/DMC = 1/1, *v*/*v*) until absorption saturation, composite gel electrolyte films were achieved. To determine their electrochemical properties, a series of batteries was assembled in an argon-encased glove box, with a stainless steel plate or lithium metal acting as the electrode, and EPS electrolyte films acting as separator as well as gel electrolyte. Ionic conductivity (*σ*) plays a vital role in assessing the performance of polymer electrolytes. Thus, we conducted AC impedance tests to calculate the *σ* of the SS|EPS|SS symmetrical battery. Figure 3a–d show the Nyquist plots and Arrhenius plots at various temperatures of EPS electrolyte films. As the temperature increases, the ionic conductivity of the battery rises, which is attributed to the lithium salts in the electrolyte accelerating decomposition and faster migration of lithium ions. The values of *σ* of EPS films at 25 °C are listed in Table 1. It can be seen that *σ* increases with the SL content. However, the highest value is only 4.4 × 10^−5^ S cm^−1^, which may be attributed to the relatively low Li-ion concentration in EPS compared with that in commercial batteries (1 M). Linear sweep voltammetry was utilized to determine the electrochemical stability range. As depicted in Figure 3e, the decomposition voltages of the batteries using EPS electrolyte films with 5 wt%, 10 wt%, and 15 wt% of SL content are 4.3 V, 4.4 V, and 4.8 V at room temperature, respectively, all higher than that required for commercial LIBs (4.2 V), indicating that the EPS electrolyte films have superior electrochemical stability. *t*_Li+_ represents the fraction of migrating lithium ions out of the total migrating ions within the electrolyte, which indicates the extent of lithium ions’ conductivity contribution to the overall ionic conductivity. In these electrolytes with low *t*_Li+_, since the anions exhibit a quicker migration rate compared to lithium ions, in the charging process there will be a concentrated polarization phenomenon.A substantial concentration of anions will accumulate at the cathode, whereas the anions at the anode will be consumed, resulting in the development of a space charge region, bringing about rapid development of lithium dendrite; thus even a minor rise of *t*_Li+_ can effectively address the aforementioned issue. As is shown in Figure 3f–h, the calculated *t*_Li+_ values are 0.53, 0.85, and 0.39 for batteries assembled with EPS films containing 5 wt%, 10 wt%, and 15 wt% of SL, respectively, indicating the best separator to inhibit lithium dendrite formation contains 10 wt% of SL. With the increase of SL content in the EPS film, the concentration of carrier becomes larger, and therefore the *σ* increases. However, excessive SL nanoparticles may accumulate and cause uneven Li ion distribution, conversely leading to the decrease of *t*_Li+_. Therefore, we chose EPS film with 10 wt% of SL content for the next experiment.

**Figure 4:**
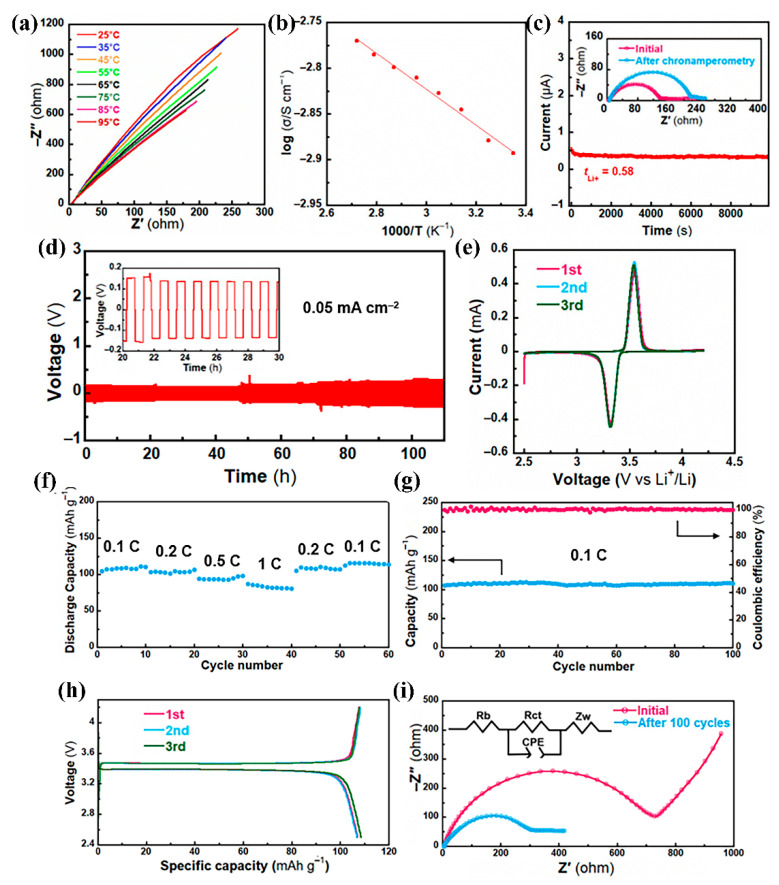
Electrochemical evaluation of EPSS-0.2 film: (**a**) Nyquist plot at various temperatures; (**b**) Arrhenius plots at various temperatures; (**c**) chronoamperometry curve with a step voltage of 10 mV at ambient temperature (inset is the corresponding EIS before and after polarization); (**d**) voltage–time profile at a current density of 0.05 mA cm^−2^ (inset is the curve between 20 and 30 h); (**e**) cyclic voltammetry curve of EPSS-0.2 film at a sweep rate of 0.1 mV s^−1^; (**f**) rate performance; (**g**) cycling performance; (**h**) galvanostatic charge/discharge plots at various cycles; (**i**) Nyquist plots before and after 100 cycles (inset is the equivalent electrical circuit).

Because of the low crystalline of P(VDF-HFP), the obtained EPS films were too soft, therefore, EPS film containing 10 wt% of SL was used to make EPSS film by covering a layer of silica onto the fibers to enhance the mechanical strength and thermal dimensional stability. The uptake of electrolyte by EPSS reaches 920%, much higher than many reported separators. According to related studies [36,37,38], even a moderate increase in *t*_Li+_ can significantly inhibit the development of lithium dendrite, and high *σ* is essential for preventing the formation of lithium dendrite in addition to high *t*_Li+_. The low lithium-ion concentration in EPSS may limit the performance of the battery. Therefore, EPSS-0.2 electrolyte film was prepared by incorporating a small amount (0.2 M) of extra LiTFSI into the plasticizer to further validate the practical utility of EPSS. As depicted in Figure 4a,b, the ionic conductivity of EPSS-0.2 has been elevated to 1.3 × 10^−3^ S cm^−1^ (25 °C). Though the corresponding *t*_Li+_ reduces to 0.58, as is shown in Figure 4c, it is still at a high level, which can effectively inhibit the concentration gradient inside the battery.

Li|EPSS-0.2|Li and LiFePO_4_|EPSS-0.2|Li batteries were assembled and their electrochemical performances were tested. A cyclic steady current with a current density of 0.05 mA cm^−2^ was utilized at both ends of the Li|EPSS-0.2|Li battery, and the stripping and plating processes were examined, as depicted in Figure 4d. The battery voltage does not increase significantly during the 110 h cycles. A stable voltage plateau can be observed in each cycle without generating overpotentials, indicating that the concentration gradient inside the battery is suppressed, which in turn inhibits the formation and development of lithium dendrite. The cyclic voltammetry curve of the LiFePO_4_|EPSS-0.2|Li battery at a sweep rate of 0.1 mV s^−1^ is shown in Figure 4e. Smooth and symmetrical redox peaks can be observed in the figure, indicating that Li ions in the battery are electrochemically deposited and dissolved well at the electrode. As is depicted in Figure 4f, the LiFePO_4_|EPSS-0.2|Li battery can work stably, with the first-cycle discharge specific capacities of 104.6, 103.0, 94.4, and 87.2 mAh g^−1^ at various rates (0.1, 0.2, 0.5, and 1 C), respectively. Upon returning the current density to 0.1 C, the battery reaches a discharge specific capacity of 112.7 mAh g^−1^. Subsequently, the LiFePO_4_|EPSS-0.2|Li battery underwent a prolonged cycling test, and the current density was 0.1 C (Figure 4g,h). The discharge specific capacity remains stable throughout the 100 cycles, and the coulombic efficiency is nearly 100%, demonstrating excellent cycling stability. The EIS impedance was measured for the battery separately before and after cycling, and is displayed in Figure 4i, where the charge transfer impedance reduces from 710.7 Ω to 327.3 Ω, which further illustrates the improved compatibility between electrolyte and electrode interface.

**Figure 5:**
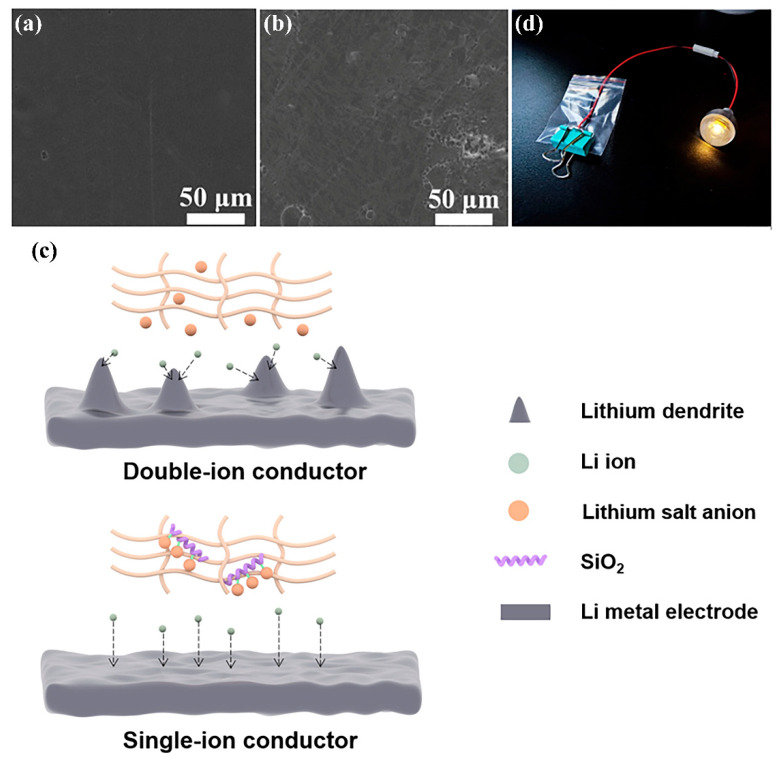
FE-SEM images showing the interface condition of (**a**) initial lithium metal anode and (**b**) that after 100 cycles; (**c**) schematic representation of lithium deposition behavior on the lithium metal anode interface of double-ionic conductor and single-ionic conductor; (**d**) photo of LiFePO_4_|EPSS-0.2|Li battery lighting LED.

The interface condition of the lithium wafers before and after charge/discharge cycling in the Li|EPSS-0.2|Li battery was compared by their FE-SEM images (Figure 5a,b). It can be seen that after 100 cycles, the interface of the lithium electrode is still smooth. Moreover, no lithium dendrite growth and uneven lithium deposition is found, which confirms the suppression of lithium dendrite development. The evident inhibitory impact of EPSS-0.2 film on the development of lithium dendrite can be rationalized as follows: in standard polymer electrolytes, Li ions are tightly associated with the Lewis basic groups found in the polymer backbone, whereas anions migrate roughly four times quicker than Li ions [39]. This discrepancy often results in a low *t*_Li+_ value, usually less than 0.5, which hinders the cycling performance of batteries. The quicker anion migration relative to Li ions causes significant polarization as the charge cycle advances, leading to an excessive build-up of anions at the cathode. At the same time, the anions at the anode become depleted, creating a space charge region. This situation can ultimately result in the rapid formation of lithium dendrite, as is shown in Figure 5c. On the contrary, in this work, the lithium salt-grafted silica nanofibers worked as the single-ion conductor. The anions were immobilized on the backbone of the silica nanofibers, which were entangled with the polymer matrix. Therefore, the concentration gradient polarization due to the anion mobility could be effectively reduced, leading to the homogeneous deposition of lithium. Though a small amount of extra lithium salt was added to the gel electrolyte film to improve the ionic conductivity, the *t*_Li+_ still reached 0.58. These optimized effects led to the good performance of EPSS-0.2 in LIBs. Moreover, the LiFePO_4_|EPSS-0.2|Li battery can successfully light up a white LED (Figure 5d), which demonstrates that this LIB can be used for practical energy storage devices.

Compared with reported polymer/ceramics composite gel electrolytes in Table 1, herein, the obtained electro-spun P(VDF-HFP)/SL composite gel electrolyte film exhibits superior comprehensive performance in LIBs, which may be attributed to its unique three-dimensional stacking nanofiber structure that forms a continuous clustered porous structure, providing high porosity and abundant pathways for lithium-ion transport. Meanwhile, the existence of a single-ion conductor increases *t*_Li+_ and suppresses the development of lithium dendrite.

## 4. Conclusions

This work presents a facile fabrication way for advanced quasi-solid electrolytes for LIBs. Lithium salt-grafted silica was incorporated into P(VDF-HFP) to make nanofiber film by the electrostatic spinning method. Meanwhile, a layer of SiO_2_ covered the nanofibers through a sol-gel method to enhance the thermal and mechanical properties of the film. Thereafter, composite gel electrolyte films were achieved by absorption of plasticizer. The obtained electrolyte film shows high thermal stability (~450 °C), high ionic conductivity (~1.3 × 10^−3^ S cm^−1^ at 25 °C) and lithium-ion transference number (0.58), and superior cycling stability. The synthesized electro-spun composite polymer electrolyte can be prepared with different sorts of polymers, and the doped lithium salt-grafted silica can be replaced with similar single-ion conductors, thereby presenting novel directions for the manufacture of safer and higher performance LIBs.

## Figures and Tables

**Table 1:** SL content, lithium-ion transference number, ionic conductivity at ambient temperature, and activation energy of four separators and several reported systems.

Separator	SL Content (%)	*t*_Li+_	*σ* (25 °C) (S cm^−1^)	*E*_a_ (KJ mol^−1^)
EPS	5	0.53	1.2 × 10^−5^	17.4
	10	0.85	2.1 × 10^−5^	13.0
	15	0.39	4.4 × 10^−5^	10.1
EPSS-0.2	10	0.58	1.3×10^−3^	3.8
P(VDF-HFP)/NMP [20]	—	0.57	4.8 × 10^−5^	—
P(VDF-HFP)/TiO_2_ [21]	—	0.53	1.2 × 10^−4^	—
P(VDF-HFP)/SiO_2_ [30]	—	∼0.9	∼10^−3^	—
PE/SiO_2_ [31]	—	—	8.4 × 10^−4^	—
P(VDF-HFP)/SiO_2_ [35]	—	0.43	1.2 × 10^−3^	—

## Data Availability

The original contributions presented in the study are included in the article, further inquiries can be directed to the corresponding author.
